# Distinct Roles of α7 nAChRs in Antigen-Presenting Cells and CD4^+^ T Cells in the Regulation of T Cell Differentiation

**DOI:** 10.3389/fimmu.2019.01102

**Published:** 2019-05-31

**Authors:** Masato Mashimo, Masayo Komori, Yuriko Y. Matsui, Mami X. Murase, Takeshi Fujii, Shiori Takeshima, Hiromi Okuyama, Shiro Ono, Yasuhiro Moriwaki, Hidemi Misawa, Koichiro Kawashima

**Affiliations:** ^1^Department of Pharmacology, Faculty of Pharmaceutical Sciences, Doshisha Women's College of Liberal Arts, Kyoto, Japan; ^2^Laboratory of Immunology, Faculty of Pharmacy, Osaka Ohtani University, Osaka, Japan; ^3^Department of Pharmacology, Faculty of Pharmacy, Keio University, Tokyo, Japan; ^4^Department of Molecular Pharmacology, Kitasato University School of Pharmaceutical Sciences, Tokyo, Japan

**Keywords:** α7 nAChR, DO11. 10 mouse, GTS-21, regulatory T cells, Th1, Th2, Th17

## Abstract

It is now apparent that immune cells express a functional cholinergic system and that α7 nicotinic acetylcholine receptors (α7 nAChRs) are involved in regulating T cell differentiation and the synthesis of antigen-specific antibodies and proinflammatory cytokines. Here, we investigated the specific function α7 nAChRs on T cells and antigen presenting cells (APCs) by testing the effect of GTS-21, a selective α7 nAChR agonist, on differentiation of CD4^+^ T cells from ovalbumin (OVA)-specific TCR transgenic DO11.10 mice activated with OVA or OVA peptide_323−339_ (OVAp). GTS-21 suppressed OVA-induced antigen processing-dependent development of CD4^+^ regulatory T cells (Tregs) and effector T cells (Th1, Th2, and Th17). By contrast, GTS-21 up-regulated OVAp-induced antigen processing-independent development of CD4^+^ Tregs and effector T cells. GTS-21 also suppressed production of IL-2, IFN-γ, IL-4, IL-17, and IL-6 during OVA-induced activation but, with the exception IL-2, enhanced their production during OVAp-induced activation. In addition, during antigen-nonspecific, APC-independent anti-CD3/CD28 antibody-induced CD4^+^ polyclonal T cell activation in the presence of respective polarizing cytokines, GTS-21 promoted development of all lineages, which indicates that GTS-21 also acts via α7 nAChRs on T cells. These results suggest 1) that α7 nAChRs on APCs suppress CD4^+^ T cell activation by interfering with antigen presentation through inhibition of antigen processing; 2) that α7 nAChRs on CD4^+^ T cells up-regulate development of Tregs and effector T cells; and that α7 nAChR agonists and antagonists could be potentially useful agents for immune response modulation and enhancement.

## Introduction

Human peripheral blood T cells and leukemic T cell lines contain substantial amounts of acetylcholine (ACh) and express the mRNA and protein for the ACh-synthesizing enzyme choline acetyltransferase (ChAT) (1–5). Similarly, expression of ChAT mRNA and/or protein has also been detected in rat and mouse T cells (4–10). Among T cell subpopulations, human and rat CD4^+^ T cells exhibit prominent ChAT mRNA expression and contain higher amounts of ACh than do CD8^+^ T cells (6, 11). Other immune cells, including B cells (6), dendritic cells (DCs) (4, 5, 12–14) and macrophages (4, 5, 14), also express ChAT mRNA and/or contain ACh. Together, these findings indicate that immune cells have the ability to synthesize ACh via ChAT.

T and B cells, DCs and macrophages all express various subtypes of muscarinic and nicotinic ACh receptors (mAChRs and nAChRs, respectively) (1–5, 15). All five mAChR subtypes (M_1_-M_5_) are expressed to some degree in most immune cells (1–5, 15). Because of the wide variety of nAChR subtypes, available data on the expression of nAChR subtypes in immune cells are not consistent (1–5, 15). That said, immune cells most commonly express mRNAs for the α2, α5, α6, α7, α9, and α10 subunits (4, 5, 12, 16–18). Furthermore, Qian et al. (18) showed that immunological activation of T cells can modify the pattern and intensity of mAChR and nAChR expression.

Among the nAChR subtypes, the role of α7 nAChR in the regulation of immune function has drawn attention in part because stimulating α7 nAChRs on macrophages suppresses the synthesis and release of tumor necrosis factor (TNF)-α, thereby protecting mice from lethal endotoxin shock induced by lipopolysaccharide (19). α7 nAChR gene-deficient (α7-KO) mice immunized with ovalbumin (OVA) exhibit significantly higher serum antigen-specific IgG_1_ concentrations than wild-type (WT) C57BL/6J mice, and, in the presence of OVA, OVA-immunized splenic cells from α7-KO mice produce greater amounts of TNF-α, interferon (IFN)-γ, and IL-6 than do those from the WT mice (20). Furthermore, α7 nAChRs enhance B cell survival in bone marrow (21) but suppress mature B cell proliferation activated via CD40-mediated pathways (22), which suggests signaling via α7 nAChRs in B cells plays a role in regulating antibody (Ab) production (23). Stimulation of α7 nAChRs using nicotine suppresses differentiation into Th1 and Th17 cells but enhances differentiation into Th2 cells of naïve CD4^+^ T cells non-specifically activated with anti-CD3/CD28 Abs (24, 25). On the other hand, Galitovskiy et al. (26) showed that nicotine acts via α7 nAChR-mediated pathways to increase the percentage of colonic regulatory T cells (Tregs) while reducing Th17 cells in oxazolone colitis, and that nicotine increases numbers of Tregs among CD4^+^ CD62L^+^ T cells non-specifically activated using anti-CD3/CD28 Abs. These findings suggest α7 nAChR signaling may modulate immune function through regulation of such T cell activities as differentiation and cytokine production.

In the present study, we endeavored to clarify the roles of α7 nAChRs expressed on T cells and antigen presenting cells (APCs) during regulation of CD4^+^ T cell differentiation. The effects of 3-[(2,4-dimethoxy)benzylidene]-anabaseine (GTS-21), a selective partial α7 nAChR agonist (27), on antigen-specific, antigen processing-dependent; antigen-specific, antigen processing-independent; and antigen-nonspecific, APC-independent CD4^+^ T cell differentiation were studied in spleen cells from OVA-specific T cell receptor (TCR) transgenic DO11.10 mice (28) and α7-KO mice. Our findings demonstrate that α7 nAChR signaling in APCs suppresses CD4^+^ T cell development by interfering with antigen presentation through inhibition of antigen processing, and that α7 nAChR signaling in CD4^+^ T cells up-regulates differentiation and proliferation into both Tregs and effector T cells.

## Materials and Methods

### Animals

OVA-specific TCR transgenic DO11.10 (H-2^d^) mice on a BALB/c background, and α7 nAChR-deficient (α7-KO) (H-2^b^) mice on a C57BL/6J background were purchased from The Jackson laboratory. C57BL/6J (H-2^b^) mice were from Japan SLC.

### CD4^+^ T Cell Culture and Differentiation

Spleen cells were prepared from mice (3–6 months old) as described previously (29) and cultured in RPMI 1640 supplemented with 10% fetal bovine serum (FBS), 50 μM 2-mercaptoethanol, 100 units/ml penicillin and 100 μg/ml streptomycin at 37°C under a humidified atmosphere with 5% CO_2_.

To examine the effects of the selective partial α7 nAChR agonist GTS-21 on CD4^+^ T cell differentiation into Tregs and effector T cells (Th1, Th2, and Th17), spleen cells (4 × 10^6^ cells) were cultured for 5 days in 24-well plates in the presence of 20 μg/ml OVA with and without GTS-21 (3–30 μM).

To examine the effects of GTS-21 on antigen processing-independent differentiation, CD4^+^ T cell differentiation was activated with 200 ng/ml OVA peptide_323−339_ (OVAp) for 5 days in the absence or presence of GTS-21 (3–30 μM) under the same experimental conditions described above.

To determine the role of α7 nAChRs expressed on CD4^+^ T cells in the regulation of CD4^+^ T cell differentiation into Tregs and effector T cells, naïve CD4^+^ T cells were isolated from spleen cells from DO11.10, α7-KO, and control WT C57BL/6J mice using a naïve CD4^+^ T cell isolation kit (130-104-453, Miltenyi Biotec) according to the manufacturer's instructions. The isolated CD4^+^ cells were cultured in 96-well plates (1 × 10^5^ cells) coated with anti-CD3 Ab (145-2C11, 5 μg/ml) in the presence of anti-CD28 Ab (37.51, 1 μg/ml), GTS-21 (3–30 μM), and the respective cytokines and Abs required for induction of each subset of effector T cell (Table 1) (30).

**Table 1:** Antibodies and cytokines used for induction of Tregs and effector T cells.

	**Tregs**	**Th1**	**Th2**	**Th17**
Anti-CD3 Ab (5 μg/ml) (plate coated)	+	+	+	+
Anti-CD28 Ab (1 μg/ml)	+	+	+	+
IL-2 (20 ng/ml)	+	+	+	+
IL-4 (100 ng/ml)			+	
IL-6 (100 ng/ml)				+
IL-12 (20 ng/ml)		+		
TGF-β (1 ng/ml)	+			+
Anti-IL-4 Ab (10 μg/ml)		+		+
Anti-IFN-γ Ab (10 μg/ml)			+	+
Anti-IL-12 Ab (10 μg/ml)			+	

*Ab, antibody; IFN, interferon; IL, interleukin; TGF, transforming growth factor; Tregs, regulatory T cells*.

### Flow Cytometry

For detection of Tregs, spleen cells were stained using FITC-conjugated anti-CD4 Ab (RM4.5, Thermo Fisher Scientific) and PE-conjugated anti-CD25 Ab (PC61.5, Thermo Fisher Scientific). After fixation and permeabilization using BD Cytofix/Cytoperm solution (BD Biosciences), the cells were further stained with APC-conjugated anti-FoxP3 (3G3, Thermo Fisher Scientific). The cells were then washed with Hanks' balanced salt solution (HBSS) supplemented with 0.1% bovine serum albumin (BSA) and 0.1% NaN_3_ and subjected to flow cytometry (CytoFLEX, Beckman Coulter). A gate was set on the lymphocytes using characteristic forward scatter (FSC) and side scatter (SSC) parameters. Isotype-matched FITC, PE and APC-conjugated mouse IgG_1_ Abs were used as controls. The acquired data was analyzed using CytExpert (Beckman Coulter).

For detection of effector T cells (Th1, Th2, and Th17 cells), spleen cells were stimulated for 1 h with 50 ng/ml phorbol 12-myristate 13-acetate and 1 μg/ml ionomycin and then with GolgiStop (BD Biosciences) for 4 h. The cells were then collected, washed with HBSS supplemented with 0.1% BSA and 0.1% NaN_3_ and stained with FITC-conjugated anti-CD4 Ab for 20 min at 4°C. The stained cells were fixed and permeabilized using BD Cytofix/Cytoperm solution and further stained with PE-conjugated anti-IFN-γ Ab (XMG1.2, Thermo Fisher Scientific) (Th1), APC-conjugated anti-IL-4 Ab (11B11, Thermo Fisher Scientific) (Th2), or anti-IL-17A Ab (eBio17B7, Thermo Fisher Scientific) (Th17) for 20 min at 4°C. After washing, the prepared cells were subjected to flow cytometry.

For cell proliferation assay, spleen cells prepared from DO11.10 mice were stained with 5 μM carboxyfluorescein succinimidyl ester (CFSE) cell proliferation reagent (Nacalai tesque) in PBS for 10 min, and cultured for 5 days under the same experimental conditions as described above with OVA or OVAp in the presence or absence of 30 μM GTS-21 and then stained with APC-conjugated anti-CD4 Ab. After washing, the prepared cells were subjected to flow cytometry.

For detection of accessory surface molecules in APCs, spleen cells prepared from DO11.10 mice were incubated for 16 h in 24-well plates (4 × 10^6^ cells) in the presence of 20 μg/ml OVA with and without GTS-21 (30 μM). To detect CD40, CD80 and MHC class II expression, spleen cells were incubated with anti-CD16/CD32 Abs (2.4G2, BD Biosciences) and then stained using FITC-conjugated anti-CD40 Ab (3/23, Biolegend), anti-CD80 Ab (16-10A1, Biolegend), or anti-MHC class II Ab (I-A/I-E, M5/114.15.2, Biolegend) along with PE-conjugated anti-CD11b (M1/70, Thermo Fisher Scientific) and APC-conjugated anti-CD11c Abs (N418, Thermo Fisher Scientific). After washing, the prepared cells were subjected to flow cytometry.

For cell viability assays, DO11.10 spleen cells prepared as described above were cultured for 5 days in 24-well plates (4 × 10^6^ cells) in the presence of 20 μg/ml OVA or 200 ng/ml OVAp, with or without GTS-21 (30 μM). At the end of the culture, the viability (7AAD exclusion) of the CD4^+^ T cells and the CD11b^+^ and CD11c^+^ cells was determined. Briefly, spleen cells were stained with APC-conjugated anti-CD4, PE-conjugated anti-CD11b and APC-conjugated anti-CD11c Abs, after which they were counterstained with 7AAD (0.25 μg/ml) and subjected to flow cytometry.

### Enzyme-Linked Immunosorbent Assay (ELISA)

Levels of IL-2, IFN-γ, IL-4, IL-6, and IL-17 in culture supernatants were quantified using a sandwich ELISA. The following pairs of capture and biotinylated detection rat anti-mouse mAbs were used: for IFN-γ, anti-IFN-γ (P4-6A2, Biolegend) and biotin-conjugated anti-IFN-γ (XMG1.2, Biolegend) Abs; for IL-2, anti-IL-2 (JES6-1A12, BD Biolegend) and biotin-conjugated anti-IL-2 (JES6-5H4, BD Biolegend) Abs; for IL-4, anti-IL-4 (11B11, Biolegend) and biotin-conjugated anti-IL-4 (BVD6-24G2, Biolegend) Abs; for IL-6, anti-IL-6 (MP5-20F3, BD Biosciences) and biotin-conjugated anti-IL-6 (MP5-32C11, BD Biosciences); for IL-17, anti-IL-17 Ab (TC11-18H10, BD biosciences) and biotin-conjugated anti-IL-17Ab (TC11-8H4, BD Biosciences). Capture Abs (2 μg/ml) were coated onto 96-well plates. After blocking with 0.5% BSA in PBS containing 0.05% Tween 20, the diluted samples and recombinant protein standards were incubated for 1 h at room temperature. Plates were then incubated with biotin-conjugated detection Abs (1 μg/ml) for 1 h at 37°C and reacted with streptavidin-conjugated horseradish peroxidase, followed by *o*-phenylenediamine. The reaction was terminated by addition of 0.5 M H_2_SO_4_. The absorbance at 490 nm was then measured, and a graph was created by analyzing three samples.

### Detection of OVA Uptake Into APCs

Spleen cells (4 × 10^6^ cells) were incubated for 4 h with FITC-labeled OVA (OVA-FITC) (50 μg/ml, Thermo Fisher Scientific) in the presence or absence of GTS-21 (30 μM). To observe OVA-FITC uptake using confocal microscopy, the cells were plated onto poly-d-lysine-coated glass-bottom dishes, fixed with 4% paraformaldehyde for 20 min at 4°C, and permeabilized and blocked with Blocking One (Nacalai tesque) containing 0.5% Triton X-100 for 1 h at room temperature. The cells were then incubated with PE-conjugated anti-CD11b and APC-conjugated anti-CD11c Abs for 20 min at 4°C. Nuclei were stained with DAPI (300 nM) for 10 min at room temperature. Cells were imaged using a confocal microscope (Zeiss LSM 800 Meta, Carl Zeiss, Inc., Germany) equipped with an oil-immersion objective (40 × , NA = 1.3). Fluorescence images were processed using ImageJ 1.37a (National Institutes of Health). For flow cytometric analysis, spleen cells were stained with PE-conjugated anti-CD11b and APC-conjugated anti-CD11c Abs for 20 min at 4°C and subjected to flow cytometry.

### Statistical Analysis

Data are presented as means ± S.E.M. All experiments were repeated at least three times. Statistical analysis was performed using SigmaPlot (Systat Software Inc.). Differences between two groups were evaluated using Student's *t*-test, and between three or more groups using one- and two-way analysis of variance (ANOVA) with *post hoc* Dunnett's or Tukey's test, respectively. Values of *P* < 0.05 were considered significant.

## Results

### Effect of GTS-21 on Antigen-Specific CD4^+^ T Cell Differentiation Induced by OVA

**Figure 1:**
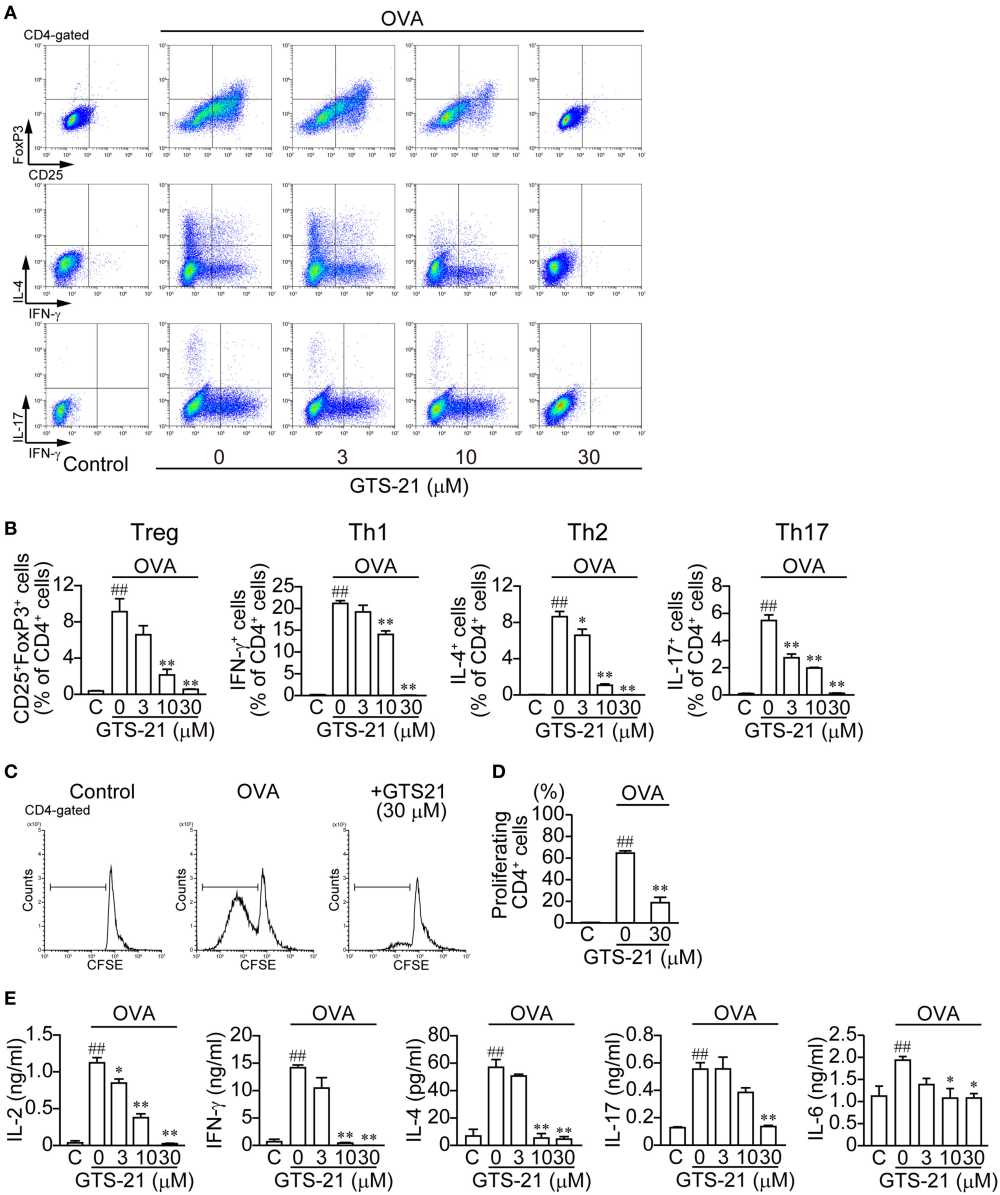
GTS-21 suppresses CD4^+^ T cell differentiation in OVA-activated DO.11.10 spleen cells. The cells were cultured with OVA and the indicated concentrations of GTS-21 for 5 days. Cultured cells were examined for surface expression of CD4, CD25 and intracellular expression of FoxP3, IL-4, IL-17, IFN-γ. Plots are gated on CD4^+^ T cells. **(A)** Representative flow cytometric plots for CD4^+^CD25^+^FoxP3^+^ T cells (Tregs), CD4^+^IFN-γ^+^ T cells (Th1), CD4^+^IL-4^+^ T cells (Th2), and CD4^+^IL-17^+^ T cells (Th17) at the indicated concentrations of GTS-21. **(B)** Corresponding percentages of OVA-activated Tregs and Th1, Th2, and Th17 cells of CD4^+^ T cells detected at the indicated concentrations of GTS-21. The bars represent means ± SEM for at least three samples. Note that GTS-21 suppressed OVA-activated differentiation. C, control (without OVA). ^##^*P* < 0.01 vs. C. *P < 0.05, ^**^P < 0.01 vs. GTS-21 at 0 μM. **(C)** Representative flow cytometric histograms for CFSE-labeled CD4^+^ cells after 5-day incubation with OVA with or without GTS-21. **(D)** Corresponding percentages of proliferating CD4^+^ cells. The bars represent means ± SEM for at least three samples. C, control (without OVA). ^*##*^*P* < 0.01 vs. C. ^**^P < 0.01 vs. GTS-21 at 0 μM. **(E)** Effect of GTS-21 on Th cytokine synthesis. Levels of IL-2, IFN-γ, IL-4, IL-17, and IL-6 in the conditioned media were determined using ELISAs after culture for 5 days. The bars represent means ± SEM for at least three samples. Note that GTS-21 suppressed OVA-activated Th cytokine synthesis. C, control (without OVA). ^##^*P* < 0.01 vs. C. ^*^P < 0.05, ^**^P < 0.01 vs. GTS-21 at 0 μM.

To activate CD4^+^ T cells and induce differentiation, OVA must be endocytosed into APCs, processed to OVAp, and bound to MHC class II molecules before presentation to CD4^+^ T cells. As shown in Figure 1, OVA (20 μg/ml) induced CD4^+^ T cell development into Tregs (CD4^+^CD25^+^FoxP3^+^) and Th1 (CD4^+^IFN-γ^+^), Th2 (CD4^+^IL-4^+^), and Th17 (CD4^+^IL-17^+^) cells (Figure 1B; Control (C) vs. OVA). GTS-21 dose-dependently suppressed OVA-induced CD4^+^ T cell development into all the lineages (Figures 1A,B). GTS-21 significantly suppressed OVA-induced CD4^+^ T cell proliferation (Figures 1C,D). OVA also increased the synthesis of IL-2, IFN-γ, IL-4, IL-17, and IL-6 in DO11.10 spleen cells (Figure 1E, Control (C) vs. GTS-21 at 0 μM), and these effects too were dose-dependently suppressed by GTS-21 (Figure 1), which is consistent with its suppression of OVA-induced CD4^+^ T cell development (Figures 1A,B).

### Effect of GTS-21 on Antigen-Specific CD4^+^ T Cell Differentiation Induced by OVAp

**Figure 2:**
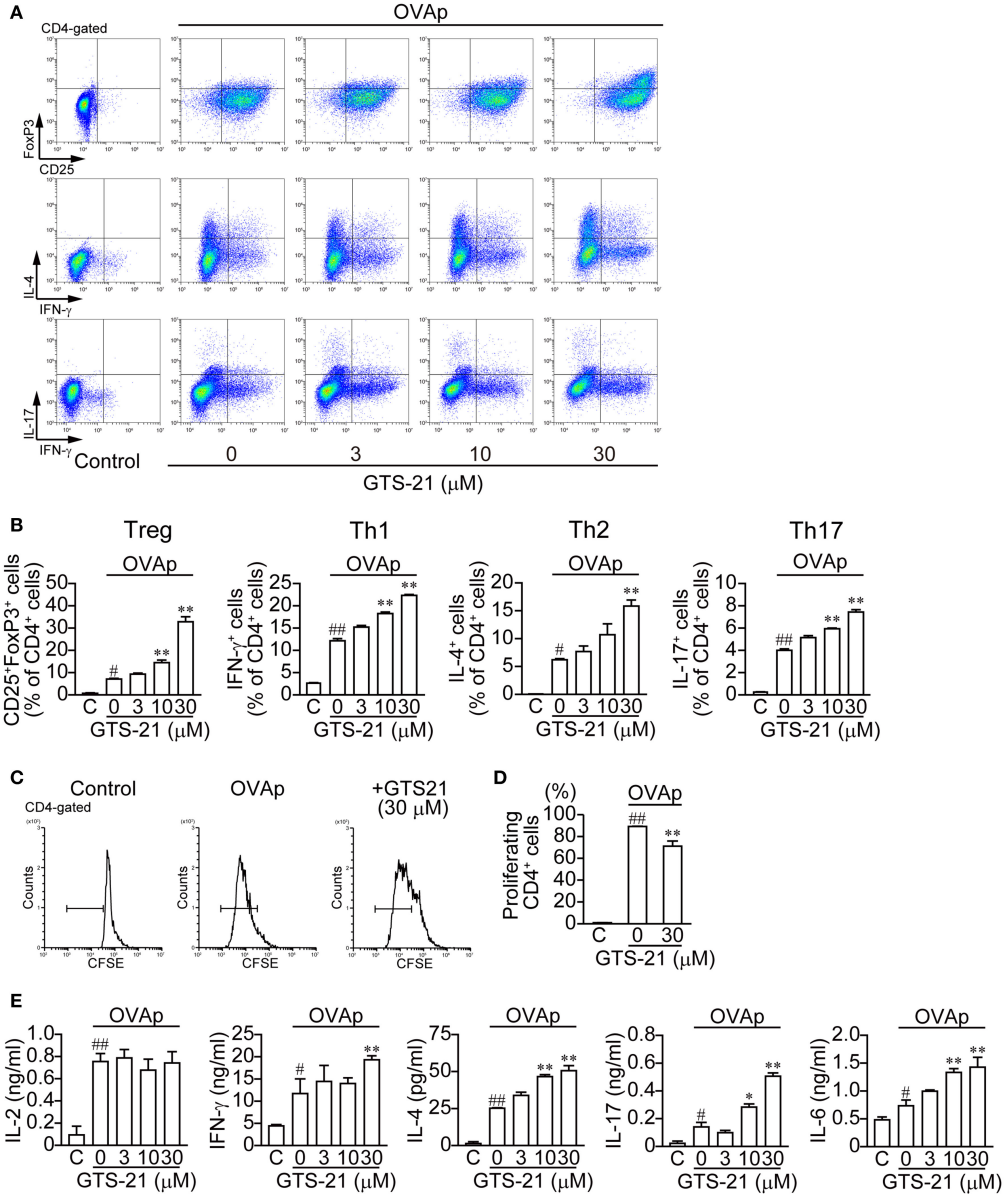
GTS-21 enhances CD4^+^ T cell differentiation in OVAp-activated DO.11.10 spleen cells. The cells were cultured with OVAp and the indicated concentrations of GTS-21 for 5 days. Cultured cells were examined for surface expression of CD4, CD25 and intracellular expression of FoxP3, IL-4, IL-17, IFN-γ. Plots are gated on CD4^+^ T cells. **(A)** Representative flow cytometric plots for Tregs, Th1, Th2, and Th17 at the indicated concentrations of GTS-21. Gates were used to calculate the percentages of positive cells. **(B)** Corresponding percentages of OVAp-activated Tregs and Th1, Th2, and Th17 cells of CD4^+^ T cells in the presence of the indicated concentrations of GTS-21. Note that GTS-21 up-regulated OVAp-activated differentiation into all lineages. The bars represent means ± SEM for at least three samples. C, control (without OVAp). ^#^*P* < 0.05, ^*##*^*P* < 0.01 vs. C. ^*^P < 0.05, ^**^P < 0.01 vs. GTS-21 at 0 μM. **(C)** Representative flow cytometric histograms for CFSE-labeled CD4^+^ cells after 5-day incubation with OVAp with or without GTS-21. **(D)** Corresponding percentages of proliferating CD4^+^ cells. The bars represent means ± SEM for at least three samples. C, control (without OVA). ^*##*^*P* < 0.01 vs. C. ^**^P < 0.01 vs. GTS-21 at 0 μM. **(E)** Effect of GTS-21 on Th cytokine synthesis. Levels of IL-2, IFN-γ, IL-4, IL-17, and IL-6 in the conditioned media were determined using ELISAs after culture for 5 days. The bars represent means ± SEM for at least three samples. Note that GTS-21 enhanced OVAp-activated synthesis of Th cytokines, except IL-2. C, control (without OVA). ^##^*P* < 0.01 vs. C. ^*^P < 0.05, ^**^P < 0.01 vs. GTS-21 at 0 μM.

In contrast to OVA, which must be taken up by APCs and processed, OVAp binds directly to MHC class II molecules on the surface of APCs to activate CD4^+^ T cell differentiation. OVAp (200 ng/ml) activated CD4^+^ T cell development into Tregs (CD4^+^CD25^+^FoxP3^+^) and Th1 (CD4^+^IFN-γ^+^), Th2 (CD4^+^IL-4^+^) and Th17 (CD4^+^IL-17^+^) cells (Figure 2). GTS-21 dose-dependently up-regulated OVAp-activated CD4^+^ T cell development into all the lineages (Figure 2). This suggests GTS-21 suppresses OVA-activated CD4^+^ T cell development by impairing APC endocytosis and/or antigen processing. GTS-21 significantly but slightly suppressed OVAp-induced CD4^+^ T cell proliferation (Figures 2C,D). OVAp also increased the synthesis of IL-2, IFN-γ, IL-4, IL-17, and IL-6 in DO11.10 spleen cells (Figure 2E, Control (C) vs. GTS-21 at 0 μM) and, with the exception of IL-2 production, those effects were enhanced by GTS-21 (Figure 2E), which reflected well its effects on OVAp-induced T cell development (Figures 2A,B). That IL-2 production was not affected by GTS-21 suggests α7 nAChRs on CD4^+^ T cells are not involved in IL-2 synthesis.

These findings shown in Figures 1, 2 suggest that GTS-21 does not affect the polarizing cytokine synthesis necessary for development of Tregs and effector T cells by spleen cells, but that GTS-21 inhibits OVA processing to OVAp in APCs, which leads to suppression of CD4^+^ T cell development.

### Effect of GTS-21 on OVA Endocytosis Into APCs and MHC Class II, CD40, and CD80 Expression

**Figure 3:**
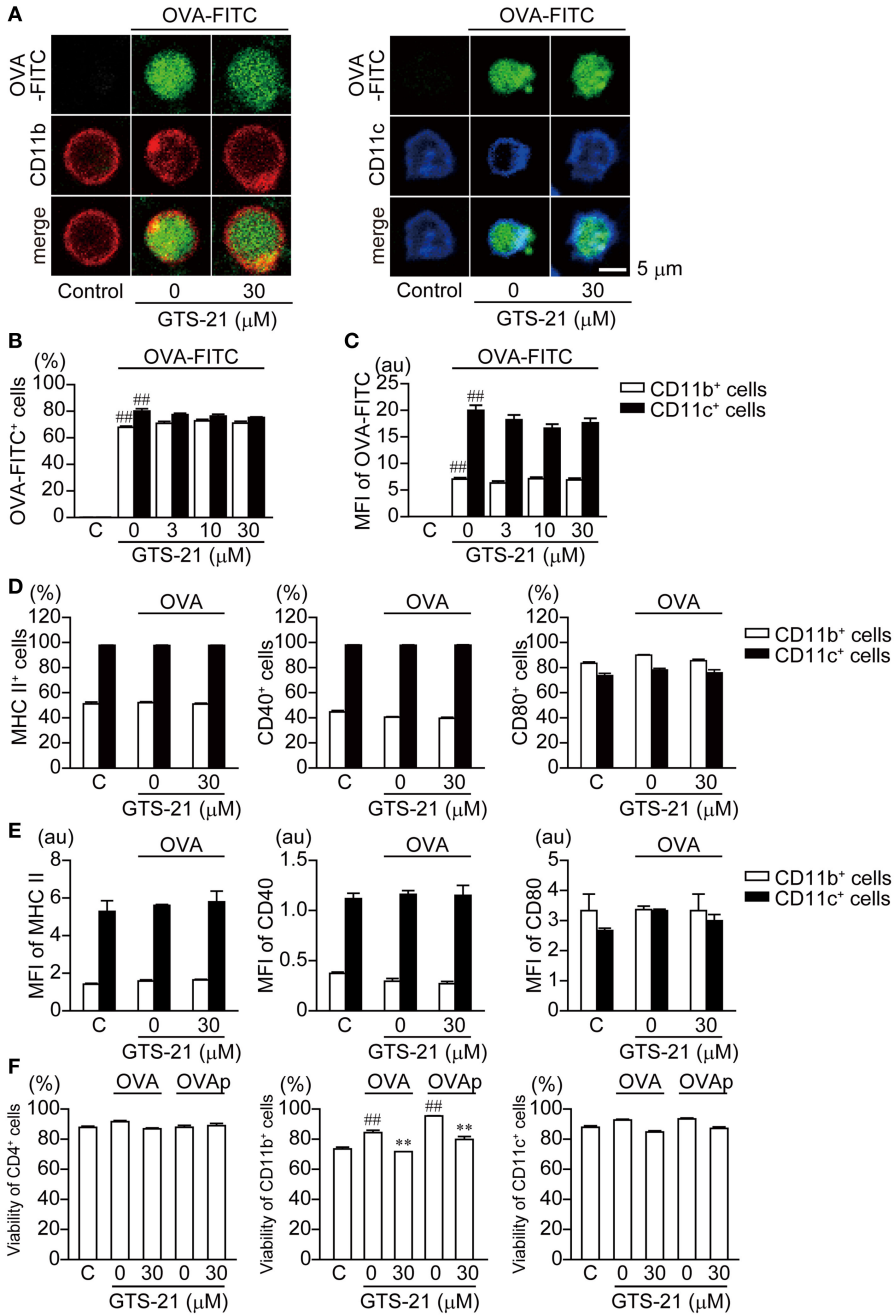
Effects of GTS-21 on FITC-OVA endocytosis; expression of MHC class II, CD40, and CD80 molecules in DO.11.10 CD11b^+^ and CD11c^+^ cells; and the viability of DO.11.10 spleen cells. **(A)** Representative micrographs showing FITC-OVA endocytosis in CD11b^+^ and CD11c^+^ cells. The spleen cells were cultured with FITC-OVA (50 μg/ml) on poly-d-lysine-coated glass-bottom dishes in the presence or absence of GTS-21 for 4 h. **(B)** Flow cytometric analysis of OVA-FITC uptake into CD11b^+^ and CD11c^+^. Graphs show the percentages of OVA-FITC^+^CD11b^+^ (left) or CD11c^+^ (right) cells in the presence of the indicated concentrations of GTS-21. The bars represent means ± SEM for at least three samples. C, control (without OVA-FITC). ^*##*^*P* < 0.01 vs. C. Note that GTS-21 did not affect FITC-OVA endocytosis in APCs. **(C)** Mean fluorescence intensity (MFI) of the gated positive population for each of the respective OVA-FITC^+^CD11b^+^ (left) or CD11c^+^ (right) markers. **(D)** Percentages of CD11b^+^ and CD11c^+^ cells showing surface expression of MHC class II, CD40, and CD80. DO.11.10 spleen cells were cultured for 16 h with OVA in the presence or absence of GTS-21 (30 μM). **(E)** MFI of MHC II, CD40 and CD80 in CD11b^+^ (left) or CD11c^+^ (right) markers. Note that GTS-21 did not affect surface expression of MHC class II, CD40 or CD80 in APCs. **(F)** Viability (7AAD exclusion) of CD4^+^ T cells, and CD11b^+^ and CD11c^+^ cells. C, control (without OVA). ^*##*^*P* < 0.01 vs. C. ^**^P < 0.01 vs. GTS-21 at 0 μM. Note that GTS-21 slightly decreased the viability of CD11b^+^ cells but had no significant effect on the viability of CD4^+^ T cells or CD11c^+^ cells.

APCs, such as macrophages (CD11b^+^) and dendritic cells (DCs, CD11c^+^), are responsible for antigen presentation to CD4^+^T cells (31). Whether GTS-21 affects the endocytosis of OVA into the CD11b^+^ and CD11c^+^ cells was investigated 4 h after addition of OVA-FITC to the cultures. Both CD11b^+^ and CD11c^+^ cells endocytosed OVA-FITC, and GTS-21 (30 μM) did not affect the fluorescent signal from these cells (Figure 3A). Moreover, flow-cytometric analysis revealed that GTS-21 did not affect the number of fluorescence-positive CD11b^+^ and CD11c^+^ cells or the mean fluorescence intensity (MFI) from CD11b^+^ and CD11c^+^ cells, indicating that α7 nAChRs are not involved in regulating OVA endocytosis into APCs (Figures 3B,C). GTS-21 also did not affect the expression of MHC class II, CD40 or CD80 molecules (Figure 3D) as well as their MFIs, indicating that α7 nAChRs are not involved in regulation of expression of these molecules on APCs (Figure 3E). These results suggest that GTS-21 impairs antigen processing after the endocytosis. On the other hand, GTS-21 did not affect the viability of CD4^+^ T or CD11c^+^ cells in the presence of OVA or OVPp, though it slightly decreased the viability of CD11b^+^ cells under both conditions, suggesting a minor role for CD11b^+^ cells as APCs for OVA antigen (Figure 3F).

### Effect of GTS-21 on Antigen-Non-Specific, APC-Independent CD4^+^ T Cell Differentiation

**Figure 4:**
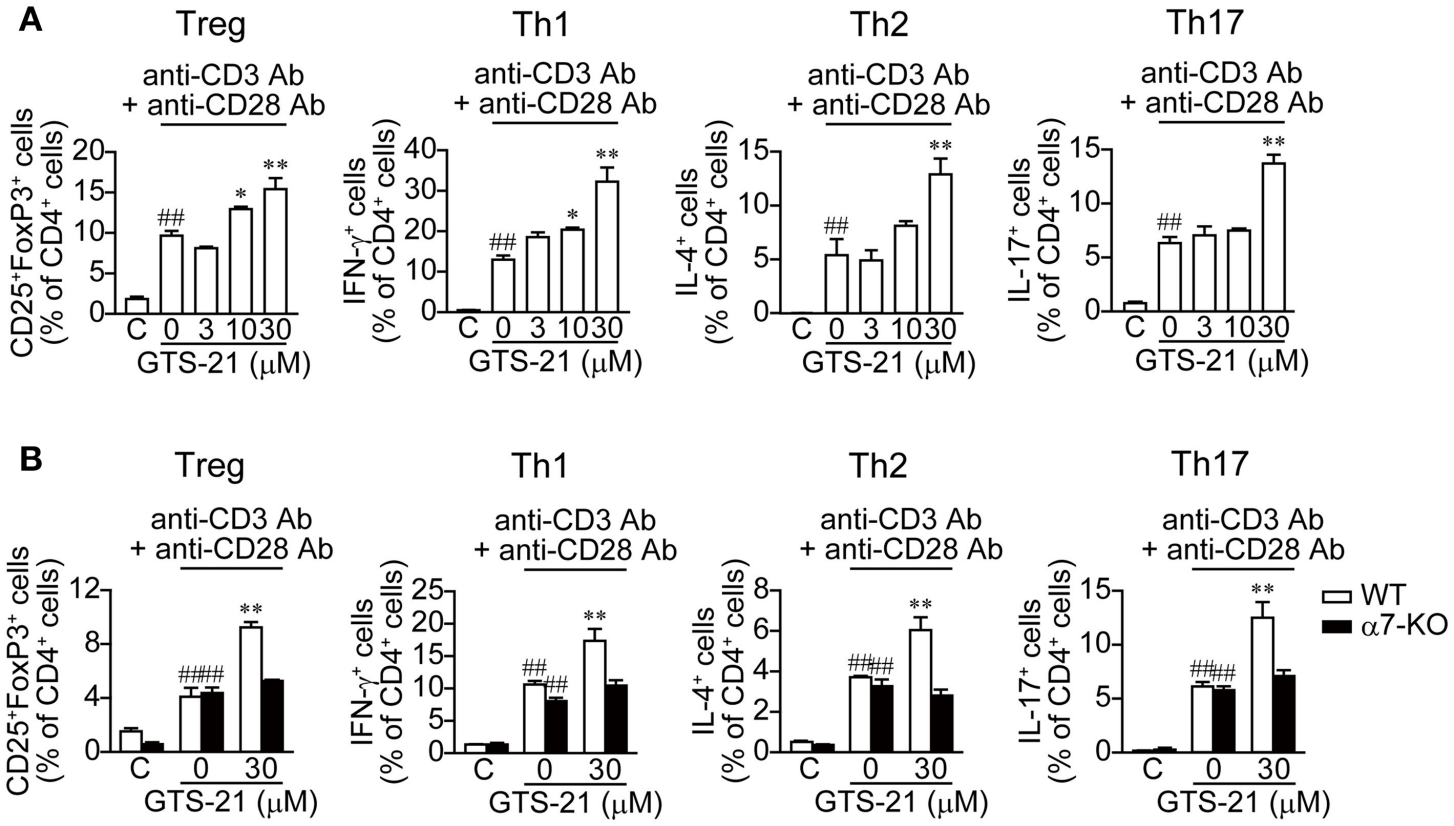
GTS-21 enhances APC-independent CD4^+^ T cell differentiation induced by anti-CD3/CD28 Abs. **(A)** Naïve CD4^+^ T cells isolated from the spleens of DO11.10 mice using a naïve CD4^+^ T cell isolation kit (130-104-453, Miltenyi Biotec) were stimulated with anti-CD3/CD28 and cultured for 5 days under Treg, Th1, Th2, and Th17 polarizing conditions (Table 1) in the presence of the indicated concentrations of GTS-21. **(B)** Naïve CD4^+^ T cells isolated from the spleens of WT or α7-KO mice were stimulated with anti-CD3/CD28 Abs and cultured for 5 days under Treg, Th1, Th2, and Th17 polarizing conditions (Table 1) in the absence or presence of 30 μM GTS-21. Note that GTS-21 enhanced T cell differentiation only in CD4^+^ cells from the WT mice. The bars represent means ± SEM for at least three samples. C, control (without OVA). ^*##*^*P* < 0.01 vs. C. ^*^P < 0.05, ^**^P < 0.01 vs. GTS-21 at 0 μM.

We next determined the roles of α7 nAChRs expressed on DO11.10 CD4^+^ T cells in the regulation of polyclonal CD4^+^ T cell differentiation activated with anti-CD3/CD28 Abs in the presence and absence of GTS-21. To induce naïve CD4^+^ T cell differentiation into Tregs and effector T cells (Th1, Th2, and Th17), culture media were supplemented with the appropriate cytokines and Abs, as described in the Materials and Methods (Table 1). GTS-21 dose-dependently up-regulated naïve CD4^+^ T cell differentiation into Tregs and effector T cells, and their proliferation (Figure 4A). These effects are in line with those observed in OVAp-activated spleen cells from DO11.10 mice (Figure 2), which is consistent with the idea that α7 nAChRs expressed on CD4^+^ T cells are involved in promoting CD4^+^ T cell development into all the lineages.

Moreover, our results obtained using naïve CD4^+^ T cells from α7-KO and WT mice confirmed the involvement of α7 nAChRs on naïve CD4^+^ T cells in the promotion of T cell differentiation and proliferation. GTS-21 (30 μM) enhanced anti-CD3/CD28 Ab-activated differentiation and proliferation of polyclonal WT naïve CD4^+^ T cells into Tregs and effector T cells, but did not affect α7-KO CD4^+^ T cell differentiation (Figure 4B). These results confirm that the action of GTS-21 on α7 nAChRs expressed on CD4^+^ T cells up-regulates the cells' differentiation and proliferation.

## Discussion

T cell activation via TCR/CD3-mediated pathways up-regulates expression of cholinergic elements such as ChAT, mAChRs and nAChRs within T cells (1–5, 18, 32, 33). Secreted lymphocyte antigen-6/urokinase-type plasminogen activator receptor-related peptide (SLURP)-1, an allosteric α7 nAChR ligand (34, 35) that has been detected in CD205^+^ DCs in the human tonsil, up-regulates ChAT mRNA expression and ACh synthesis in T cells (36). This suggests ACh released from T cells and from DCs along with SLURP-1 during immune responses such as antigen presentation contributes to the regulation of immune function via α7 nAChR-mediated pathways.

DO11.10 CD4^+^ T cell differentiation is triggered by TCR recognition of OVA peptide_326−339_ (OVAp) presented on MHC class II in APCs (28). Thus, OVA activates CD4^+^ T cell differentiation only after it has been endocytosed into APCs; cleaved to OVAp by endosomal and lysosomal enzymes such as γ-interferon-inducible lysosomal thiol reductase (37, 38) and the cathepsins (39); and then translocated to the cell surface as part of an OVAp-MHC class II complex (see Supplementary Figure 1). By contrast, OVAp can directly bind to MHC class II on the surface of APCs and activate CD4^+^ T cells differentiation with no further processing (40, 41).

In the present study, GTS-21 suppressed OVA-activated DO.11.10 CD4^+^ T cell development into Tregs and effector T cells (Th1, Th2 and Th17) (Figure 1) and also suppressed the cytokine synthesis related to CD4^+^ T cell development (Figure 1E). The importance of α7 nAChRs expressed on APCs, including macrophages, to the regulation of immune function is further supported by the finding that galantamine, an acetylcholinesterase inhibitor with positive allosteric modulator activity toward α7 nAChR (42), suppressed release of IgG, IL-4, and IL-6 during an *ex vivo* antigen challenge in spleen cells from immunized mice (43). GTS-21 did not affect endocytosis of OVA or expression of MHC class II molecules by APCs (Figures 3A–D). Although GTS-21 (30 μM) slightly decreased the viability of CD11b^+^ cells in the presence of antigens, it did not affect the viability of CD4^+^ T cells or CD11c^+^ cells determined with 7AAD (Figure 3E). No apparent cytotoxicity of GTS-21, even at 50 μM, was demonstrated by Sitapara et al. (44), who reported that by inhibiting the release of nuclear HMGB1, GTS-21 at 25–50 μM restored hyperoxia-compromised particle phagocytic activity in murine macrophage-like RAW 364.7 cells. Furthermore, in the present study, GTS-21 suppressed OVA-induced APC-dependent and antigen processing-dependent CD4^+^ T cell development (Figure 1) but promoted OVAp-induced APC-dependent and antigen processing-independent CD4^+^ T cell development at the same concentrations (Figure 2). Taken together, the findings suggest GTS-21 suppresses CD4^+^ T cell development by pharmacologically inhibiting OVA antigen processing via α7 nAChRs expressed on APCs, not by eliciting functional impairments ascribable to its cytotoxicity in CD4^+^ T cells or dendritic cells (Supplementary Figures 1, 2).

GTS-21-induced up-regulation of CD4^+^ T cell development into Tregs and effector T cells in both OVAp-activated DO.11.10 CD4^+^ T cells and anti-CD3/CD28 Abs-activated CD4^+^ T cells (Figures 2, 4) suggests α7 nAChRs on CD4^+^ T cells play role in the up-regulation of T cell differentiation and proliferation. That idea is consistent with our present (Figure 4B) and earlier observations that GTS-21 up-regulates anti-CD3/CD28 Abs-activated CD4^+^ T cell development into Tregs in WT C57BL/6J spleen cells but not α7-KO spleen cells (4). The results of cell proliferation assay with CSFE suggest the possibility that GTS-21 rather up-regulates differentiation than proliferation (Figure 2C).

The T-cell activator phytohemagglutinin (PHA) up-regulates mRNA expression of IL-2 and ChAT, an enzyme catalyzing ACh synthesis. PHA most likely elicits this response by activating PKC and mitogen-activated protein kinase pathways via TCR/CD3-mediated pathways (45). Recently, Mashimo et al. (29) confirmed that ACh released from activated T cells via TCR/CD3-mediated pathways induces Ca^2+^ signaling and enhances IL-2 release leading to up-regulation of T cell proliferation, and that both IL-2 production and T cell proliferation are suppressed by mecamylamine, a non-specific nAChR antagonist. However, involvement of α7 nAChRs expressed on T cells in the induction of Ca^2+^ signaling and enhancement of IL-2 release is unlikely because those receptors are insensitive to methyllycaconitine and α-bungarotoxin, two specific antagonists that block α7 nAChRs expressed on neurons (46). This suggests nAChRs other than α7 nAChR contribute to the induction of Ca^2+^ signaling and enhanced IL-2 release in TCR/CD3-activated T cells. The induction of IL-2 production in the presence of OVA or OVAp observed in the present study (Figure 2D) can be attributed to naïve T cell activation via TCR/CD3-mediated pathways as a result of antigen presentation triggering ACh synthesis (1–5). Thus, GTS-21 suppressed OVA-induced IL-2 production by interfering with antigen processing, which led to inhibition of antigen presentation but did not affect OVAp-induced IL-2 production, because GTS-21 does not affect OVAp presentation.

The detailed mechanisms involved in the promotion of CD4^+^ T cell development into Tregs and effector T cells via α7 nAChR-mediated pathways are still unclear. Stein and Singer (47) suggested that IL-6 could replace the requirement for APC-derived co-stimulatory signals for primary CD4^+^ Th cell proliferation. GTS-21-induced increases in IL-6 production during OVAp-activation, suggesting IL-6 is involved in enhancing CD4^+^ T cell development (Figure 2). This in turn suggests GTS-21 may affect CD4^+^ T cell development by enhancing IL-6 synthesis in CD4^+^ T cells and APCs during the early stages of differentiation. In fact, Eto et al (48) reported that optimal T cell differentiation requires IL-6 along with IL-21. IL-6 signaling in T cells is transduced through the Janus kinase (JAK) family of proteins, culminating in signal transducer and activator of transcription 3 (STAT3) activation (49, 50). STAT3 is a critical positive regulator of T cell differentiation and functions in several CD4^+^ T cell subsets, including Th2 and Th17 cells and Tregs (51–56). In non-neuronal cells such as macrophages (57) and keratinocytes (58, 59), stimulation of α7 nAChRs by agonists such as GTS-21 and SLURP-1 activates the catalytic intracellular domain of the receptor, leading to recruitment and phosphorylation of JAK2 and subsequent activation of STAT3. This suggests that in addition to promoting IL-6 synthesis, GTS-21 binding to α7 nAChRs on CD4^+^ T cells promotes their development into Tregs and effecter T cells through activation of the JAK2/STAT3 signaling cascade.

Our observation that GTS-21 suppressed antigen-specific, antigen processing-dependent CD4^+^ T cell development while promoting antigen-specific, antigen processing-independent CD4^+^ T cell development suggests α7 nAChR agonists or antagonists could potentially be useful in the treatment of autoimmune diseases or cancers. Studies of the effects of adoptive transfer of antigen-specific Tregs have demonstrated their contribution to the protection and recovery of an animal model of autoimmune encephalomyelitis (60). However, the difficulty in achieving adequate numbers of antigen-specific Tregs for adoptive transfer is a major limitation of its clinical application. The promotion of antigen-specific, antigen processing-independent CD4^+^ T cell development into Tregs by GTS-21 suggests the possibility that culturing peripheral blood mononuclear cells containing CD4^+^ T cells and APCs in the presence of specific antigenic epitopes and an α7 nAChR agonist would enhance CD4^+^ T cells development into antigen-specific Tregs, making them available in greater numbers. Moreover, because GTS-21 promotes antigen-specific, antigen processing-independent CD4^+^ T cell development into effector T cells, it may be possible to obtain sufficient numbers of the relevant subset of effector T cells for the purpose of immune enhancement.

In summary, the results of the present study revealed that activation of α7 nAChRs on APCs suppresses antigen-specific, antigen processing-dependent CD4^+^ T cell development by suppressing antigen processing. By contrast, activation of α7 nAChRs expressed on CD4^+^ T cells up-regulates antigen-specific, antigen processing-independent CD4^+^ T cell development into Tregs and effector T cells, most likely via activation of JAK2/STAT pathways. α7 nAChRs expressed by immune cells are thus crucially involved in the regulation of both innate and adoptive immunity.

## Ethics Statement

This study was carried out in accordance with the recommendations of the Ethical Committees of Doshisha Women's College of Liberal Arts. The protocol was approved by the Ethical Committees of Doshisha Women's College of Liberal Arts (Nos. Y15012, Y15028, Y16002, Y17010, Y17024, Y18001, Y18010).

## Author Contributions

KK, TF, MM, and SO were involved in study design, interpretation of the results, and writing and revising the manuscript. MM, MK, YYM, MXM, TF, ST, and HO performed experiments. YM and HM provided experimental materials and were involved in revising the manuscript. KK, TF, MM, MK, YYM, MXM, ST, YM, HM, SO, and HO reviewed and approved the final version of the manuscript.

### Conflict of Interest Statement

The authors declare that the research was conducted in the absence of any commercial or financial relationships that could be construed as a potential conflict of interest.

## Supplementary Material

Supplementary Figure 1 Schematic drawing of OVA-, OVAp-, and anti-CD3/CD28 Abs-activated CD4^+^ T cell differentiation into Tregs and effector T cells (Th1, Th2, and Th17). **(A)** OVA-activated CD4^+^ T cell differentiation of DO11.10 spleen cells. OVA endocytosed in antigen presenting cells (APCs) is processed within endosomes by lysosomal enzymes such as γ-interferon-inducible lysosomal thiol reductase (GILT) and cathepsins to OVAp. After binding to MHC class II molecules (MHC II), OVAp-MHC II complex is translocated to the surface of APCs. Recognition of OVAp presented on MHC II by TCR on DO11.10 CD4^+^ T cells generates “Signal 1” and triggers a series of activation processes leading to differentiation. Along with Signal 1, the interaction with CD80/CD86 co-stimulatory molecules via CD28 (“Signal 2”) enhances the differentiation. Thus, Signals 1 and 2 promote CD4^+^ T cell differentiation into Tregs and effector T cells. **(B)** OVA-activated differentiation of DO11.10 spleen cells in the presence of GTS-21. GTS-21 suppresses OVA-activated CD4^+^ T cell differentiation (Figure 1), most likely by suppressing lysosomal enzyme expression, and thus antigen processing, via stimulation of α7 nAChRs on APCs. This inhibition of antigen processing suppresses antigen presentation and, thus, Signal 1, which triggers CD4^+^ T cell differentiation. **(C)** OVAp-activated diffrentiation of DO11.10 spleen cells in the presence of GTS-21. OVAp activated CD4^+^ T cell development into Tregs and Th1, Th2, and Th17 (Figure 2, GTS-21 at 0 μM). OVAp directly binds to MHC II on the surface of APCs and is then recognized by TCRs on DO11.10 CD4^+^ T cells, leading to generation of Signals 1 and 2. **(D)** Anti-CD3/CD28 Abs-activated differentiation of DO11.10 naïve CD4^+^ T cells in the presence of polarizing cytokines and GTS-21. Anti-CD3/CD28 Abs activates naïve CD4^+^ T cell differentiation into Tregs and Th1, Th2, and Th17 by binding to CD3 and CD28, which leads to generation of Signals 1 and 2 (Figure 4; GTS-21 at 0 μM). GTS-21 enhances both OVAp- and Anti-CD3/CD28 Abs-activated differentiation and proliferation of Tregs and Th1, Th2, and Th17 by stimulating CD4^+^ T cell α7 nAChRs (Figure 4; GTS-21 at 30 μM).

Supplementary Figure 2 Contribution of α7 nAChRs expressed on APCs (CD11b^+^ and CD11c ^+^ cells) to the regulation of CD4^+^ T cell development. To define the role for α7 nAChRs expressed on APCs, naïve CD4^+^ T cells isolated from OVA-specific TCR transgenic OT-II (H-2^b^) mice were co-cultured for 5 days with APCs isolated from control WT C57BL/6J or α7-KO mice in the presence of 20 μg/ml OVA with and without GTS-21 (30 μM). **(A)** Representative flow cytometric plots for CD4^+^CD25^+^FoxP3^+^ T cells (Tregs). **(B)** Corresponding percentages of OVA-activated Tregs in the presence or absence of GTS-21. Note that OVA induced development into Tregs in both the WT and α7-KO samples, and that GTS-21 suppressed OVA-activated development only in the WT samples. These results suggest that activation of α7 nAChRs expressed on APCs down-regulates antigen presentation, which is a common stimulus for induction of naïve CD4^+^ T cell activation. Thus, GTS-21 also appears to suppress OVA-activated naïve CD4^+^ T cell development into effector T cells via α7 nAChRs on APCs. The bars represent means ± SEM for at least three samples. C, control (without OVA).^##^*P* < 0.01 vs. C. ^**^*P* < 0.01 vs. GTS-21 at 0 μM.
