# Supplementary figures and images for: Distinct Roles of α7 nAChRs in Antigen-Presenting Cells and CD4+ T Cells in the Regulation of T Cell Differentiation
Source: Front Immunol. 2019 May 31;10:1102. doi: 10.3389/fimmu.2019.01102 (PMC6554293; doi:10.3389/fimmu.2019.01102)

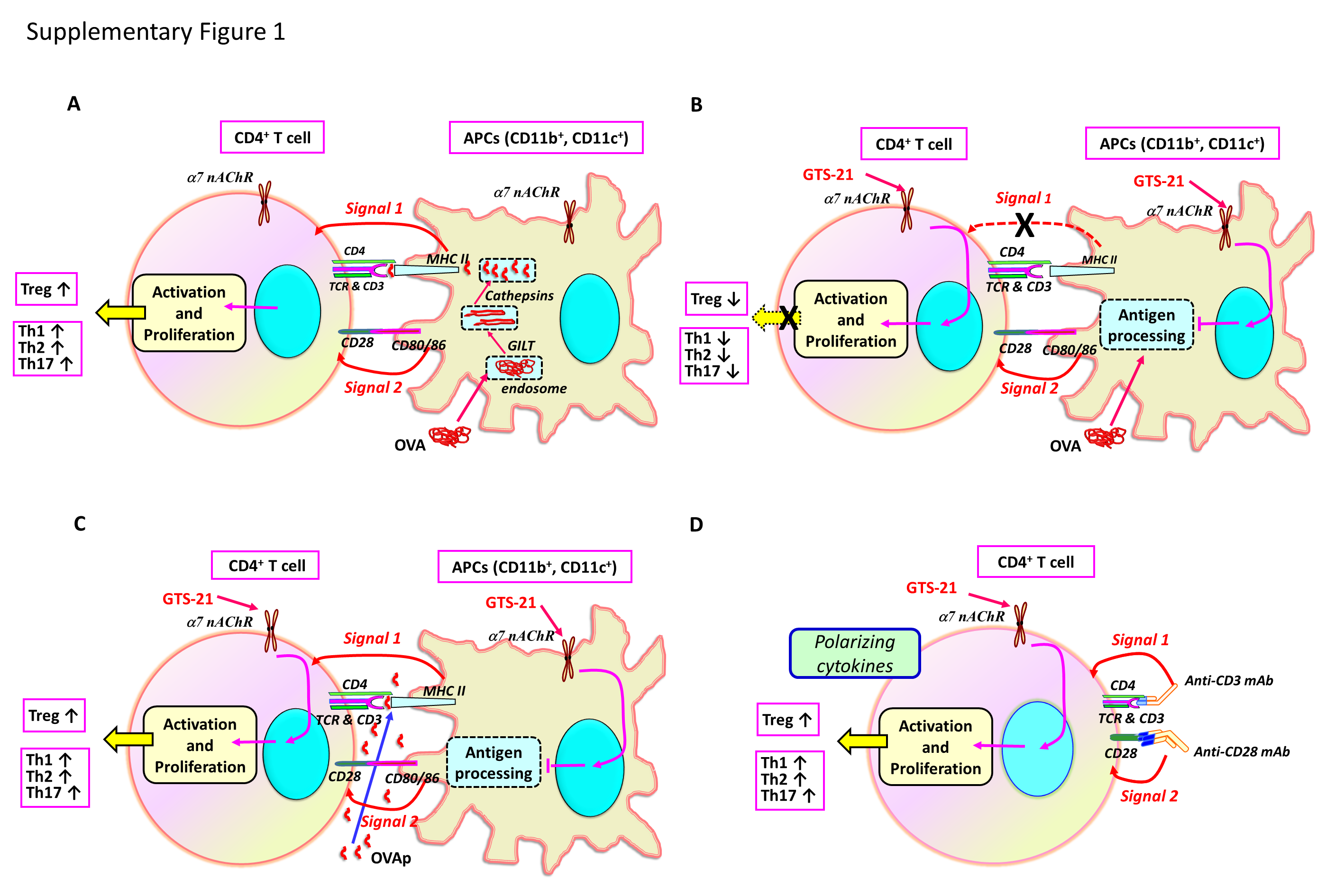

Supplement: Supplementary Figure 1 — Schematic drawing of OVA-, OVAp-, and anti-CD3/CD28 Abs-activated CD4+ T cell differentiation into Tregs and effector T cells (Th1, Th2, and Th17). (A) OVA-activated CD4+ T cell differentiation of DO11.10 spleen cells. OVA endocytosed in antigen presenting cells (APCs) is processed within endosomes by lysosomal enzymes such as γ-interferon-inducible lysosomal thiol reductase (GILT) and cathepsins to OVAp. After binding to MHC class II molecules (MHC II), OVAp-MHC II complex is translocated to the surface of APCs. Recognition of OVAp presented on MHC II by TCR on DO11.10 CD4+ T cells generates “Signal 1” and triggers a series of activation processes leading to differentiation. Along with Signal 1, the interaction with CD80/CD86 co-stimulatory molecules via CD28 (“Signal 2”) enhances the differentiation. Thus, Signals 1 and 2 promote CD4+ T cell differentiation into Tregs and effector T cells. (B) OVA-activated differentiation of DO11.10 spleen cells in the presence of GTS-21. GTS-21 suppresses OVA-activated CD4+ T cell differentiation (Figure 1), most likely by suppressing lysosomal enzyme expression, and thus antigen processing, via stimulation of α7 nAChRs on APCs. This inhibition of antigen processing suppresses antigen presentation and, thus, Signal 1, which triggers CD4+ T cell differentiation. (C) OVAp-activated diffrentiation of DO11.10 spleen cells in the presence of GTS-21. OVAp activated CD4+ T cell development into Tregs and Th1, Th2, and Th17 (Figure 2, GTS-21 at 0 μM). OVAp directly binds to MHC II on the surface of APCs and is then recognized by TCRs on DO11.10 CD4+ T cells, leading to generation of Signals 1 and 2. (D) Anti-CD3/CD28 Abs-activated differentiation of DO11.10 naïve CD4+ T cells in the presence of polarizing cytokines and GTS-21. Anti-CD3/CD28 Abs activates naïve CD4+ T cell differentiation into Tregs and Th1, Th2, and Th17 by binding to CD3 and CD28, which leads to generation of Signals 1 and 2 (Figure 4; GTS-21 at 0 μM). GTS-21 enhances [file Image_1.TIF]

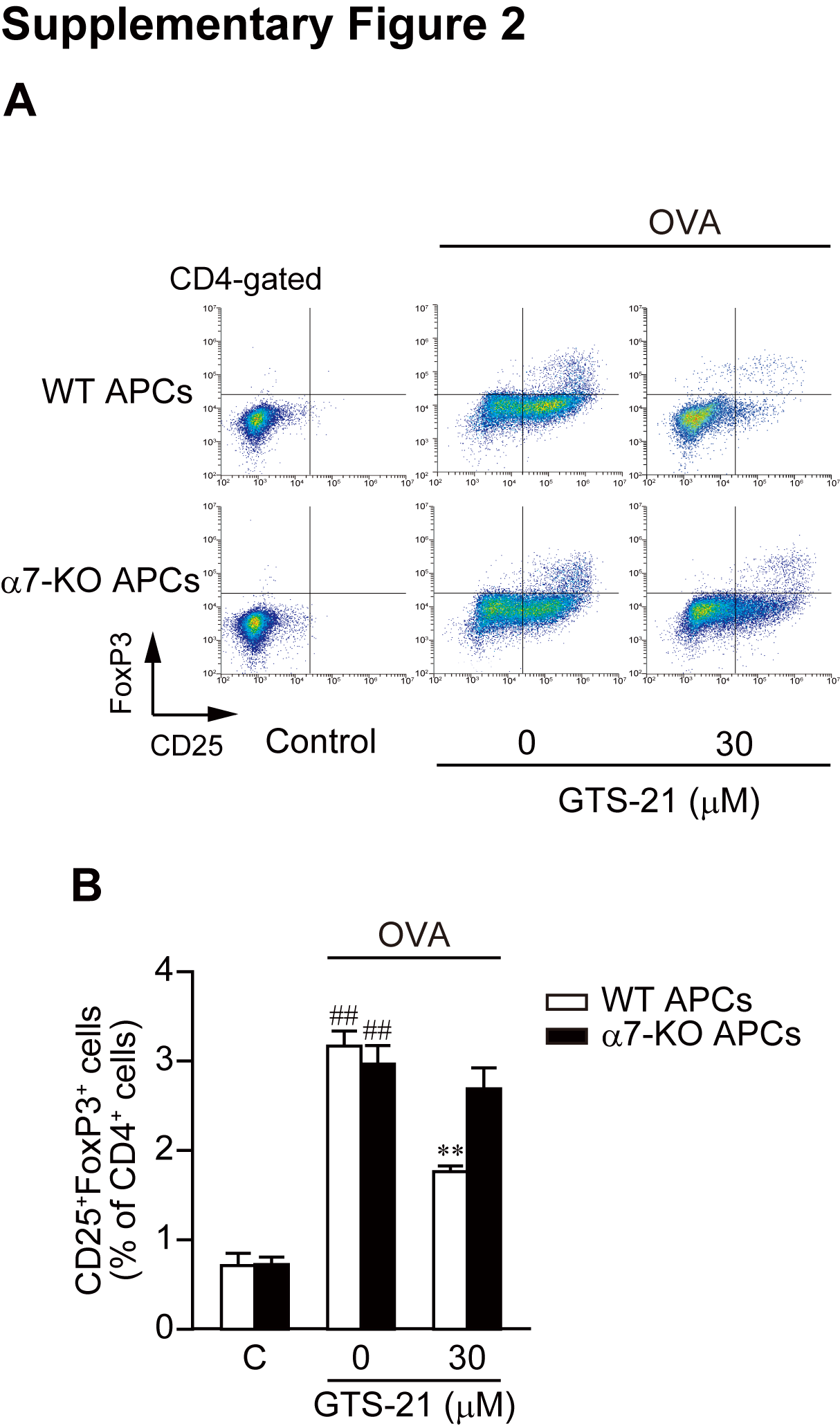

Supplement: Supplementary Figure 2 — Contribution of α7 nAChRs expressed on APCs (CD11b+ and CD11c + cells) to the regulation of CD4+ T cell development. To define the role for α7 nAChRs expressed on APCs, naïve CD4+ T cells isolated from OVA-specific TCR transgenic OT-II (H-2b) mice were co-cultured for 5 days with APCs isolated from control WT C57BL/6J or α7-KO mice in the presence of 20 μg/ml OVA with and without GTS-21 (30 μM). (A) Representative flow cytometric plots for CD4+CD25+FoxP3+ T cells (Tregs). (B) Corresponding percentages of OVA-activated Tregs in the presence or absence of GTS-21. Note that OVA induced development into Tregs in both the WT and α7-KO samples, and that GTS-21 suppressed OVA-activated development only in the WT samples. These results suggest that activation of α7 nAChRs expressed on APCs down-regulates antigen presentation, which is a common stimulus for induction of naïve CD4+ T cell activation. Thus, GTS-21 also appears to suppress OVA-activated naïve CD4+ T cell development into effector T cells via α7 nAChRs on APCs. The bars represent means ± SEM for at least three samples. C, control (without OVA).##P < 0.01 vs. C. **P < 0.01 vs. GTS-21 at 0 μM. [file Image_2.TIF]
